# Histopathological, immunohistochemical and imaging features of bone metastases of mammary carcinoma in bitches: cases report

**DOI:** 10.29374/2527-2179.bjvm009124

**Published:** 2025-03-19

**Authors:** Ana Paula Vargas Garcia, Fernanda Rezende Souza, Luiz Flávio Telles, Antônio Carlos Lacreta, Mary Suzan Varaschin, Geovanni Dantas Cassali

**Affiliations:** 1 Laboratório de Patologia Comparada, Universidade Federal de Minas Gerais, Belo Horizonte, MG, Brazil; 2 Setor de Patologia Veterinária, Universidade Federal de Lavras, Lavras, MG, Brazil

**Keywords:** canine mammary carcinoma, adenomyoepithelioma, bone metastasis in dogs, distant metastasis, bone remodeling, carcinoma mamário canino, adenomioepitelioma, metástase óssea em cães, metástase à distância, remodelação óssea

## Abstract

In the present case report, the histopathological and immunohistochemical characteristics of the two cases of bone metastasis of mammary carcinoma in bitches are described. The animal in the first case is a 10 years old female poodle. The physical examination revealed a mass in the left abdominal caudal (M4) and inguinal (M5) mammary glands with a six-month evolution. The imaging exams of the right pelvic limb revealed areas of bone lysis in the distal portion of the femur. No evidence of metastases was observed in the thorax on thoracic radiographs. Microscopic evaluations were consistent with the diagnosis of malignant adenomyoepithelioma. The mass in the distal region of the femur has characteristics similar to those observed in the mammary gland mass. The animal in the second case was a nine-year-old female mixed-breed euthanized due to the unfavorable prognosis of the disease. Histopathological evaluation of the primary tumor in M3, M4, and M5 was consistent with the diagnosis of grade II cribriform carcinoma. Metastatic foci were observed in the lung, liver, kidney, adrenal, proximal metaphyseal region of the right humerus extending to the distal diaphyseal region, and axillary and medial iliac lymph nodes’ parenchyma. Immunohistochemistry was performed for markers Ki67, Cox-2, ER, PR, Pan-CK, p63 and HER-2 in the primary tumor and bone metastasis in both cases. High proliferation rate, positivity for hormone receptors, Pan-CK and p63 were observed in both cases. HER-2 was negative in the primary tumor and bone metastasis and COX-2 was negative in the primary tumor of both cases, negative in the metastasis of case 01 and positive in the metastasis of case 02.

## Resumo

Neste relato de caso, são descritas as características histopatológicas e imunohistoquímicas de dois casos de metástase óssea de carcinoma mamário em cadelas. No primeiro caso, trata-se de um poodle, fêmea, 10 anos. O exame físico revelou uma massa nas glândulas mamárias abdominal caudal esquerda (M4) e inguinal (M5) com evolução de seis meses. Os exames de imagem do membro pélvico direito mostraram áreas de lise óssea na porção distal do fêmur. Não havia evidência de metástases no tórax nas radiografias torácicas. As avaliações microscópicas foram consistentes com o diagnóstico de adenomioepitelioma maligno. A massa na região distal do fêmur apresentou características semelhantes às observadas na massa da glândula mamária. O animal do segundo caso era uma fêmea, SRD, nove anos que foi sacrificada devido ao prognóstico desfavorável da doença. A avaliação histopatológica do tumor primário em M3, M4 e M5 foi consistente com o diagnóstico de carcinoma cribriforme grau II. Focos metastáticos foram observados no pulmão, fígado, rim, adrenal, região metafisária proximal do úmero direito estendendo-se para a região diafisária distal e parênquima linfonodal axilar e ilíaco medial. Foi realizada imunohistoquímica para os marcadores Ki67, Cox-2, ER, PR, Pan-CK, p63 e HER-2 no tumor primário e na metástase óssea em ambos os casos. Na avaliação, os casos mostraram alta taxa de proliferação, positividade para receptores hormonais, Pan-CK e p63. HER-2 foi negativo no tumor primário e na metástase óssea, e COX-2 foi negativo no tumor primário de ambos os casos, negativo na metástase do caso 01 e positivo na metástase do caso 02.

## Introduction

Mammary tumors, which are the most common neoplasms in bitches, represent a significant concern in veterinary medicine (Nunes et al., 2018). These tumors are frequently diagnosed in dogs and are the most prevalent type of tumor in bitches (Cassali et al., 2020; Nunes et al., 2018). The majority of mammary neoplasms are malignant and can be associated with increased mortality (Cassali et al., 2020; Misdorp et al., 1999; Nunes et al., 2018). This scenario underscores the current concern among veterinary oncologists regarding the importance of accurate diagnosis and effective therapeutic strategies for malignant mammary tumors (Cassali et al., 2020). The prevalence of mammary neoplasms varies significantly across different countries, primarily due to cultural differences in neutering practices (Nunes et al., 2018).

Histologically, approximately 85% of canine mammary neoplasms are classified as malignant, with distant metastases being the primary cause of death. Several clinical and pathological factors are associated with a worse prognosis, including tumor size greater than 3.0 cm, presence of undifferentiated, ulcerated, or adherent tumors. Furthermore, tumors diagnosed as solid, anaplastic, inflammatory carcinomas, or sarcomas, along with the presence of regional or distant metastases, vascular or lymphatic invasion, absence of hormone receptor expression, and high proliferation rates are also related to a poor prognosis (Cassali et al., 2020; Nunes et al., 2018; Sorenmo et al., 2013).

Bone metastases are rare in bitches with tumors; however, this occurrence may be underestimated (Cooley & Waters, 1998). When bone metastases do arise from mammary carcinomas and other tissues in dogs, they typically develop in the axial skeleton and the proximal regions of long bones, with a preference for spreading via the hematogenous route (Tahara et al., 2019). Therefore, a more detailed description of the histopathological and immunohistochemical features of bone metastases originating from mammary tumors in dogs could enhance the accuracy of diagnosis in such cases. Consequently, the aim of this study is to describe the histopathological and immunohistochemical characteristics of two cases of mammary gland neoplasms in dogs.

## Case description

Tissue samples from two female dogs were collected for histopathological and immunohistochemical analysis at the Laboratory of Comparative Pathology, Federal University of Minas Gerais, and the Veterinary Pathology Sector, Federal University of Lavras.

**Figure 1:**
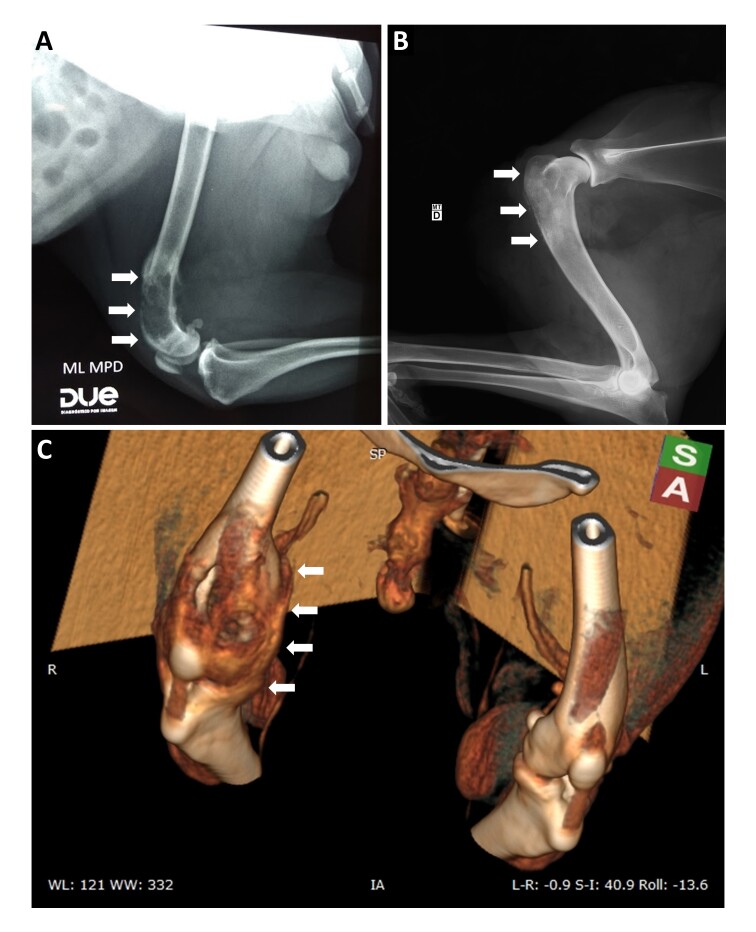
(A) Radiography of the right pelvic limb of case 01; (B) radiography of the right thoracic limb of case 02 and (C) computed tomography of the right pelvic limb of the case 01. Female dog. (A) Aggressive bone response characterized by a geographic area mainly composed of osteolysis, with poorly defined margins, cortical thinning and destruction, and amorphous, spiculated periosteal proliferation, extending from the distal metaphysis to the proximal diaphysis of the femur; (B) Moderately aggressive bone response with poorly defined medullary bone sclerosis, interrupted fine brush-like periosteal proliferation, and cortical thinning and destruction, from the proximal metaphysis to the distal diaphysis of the humerus; (C) Mixed lesion with lytic and proliferative characteristics, leading to significant cortical loss, bone matrix lysis, and invasion into the spongy bone and endosteum, located in the distal diaphysis of the right femur, extending to its distal metaphysis. Notable muscle atrophy suggesting limb disuse/lameness. No lesions observed in femoral condyles.

The first dog (case 01) was a 10-year-old female poodle that presented right pelvic limb lameness, local pain, fever, apathy, prostration, hyporexia, and progressive cachexia. The dog had a regular estrous cycle with estrus occurring every six months and no history of pregnancy, pseudocyesis, or contraceptive use. During physical examination, a mass measuring approximately 7.0 x 3.0 cm was identified in the left caudal abdominal (M4) and inguinal (M5) mammary glands, along with a slight enlargement of the left inguinal lymph node. Additionally, a non-ulcerated mass was adhered to the left humeroscapular region, with a six-month history of progression. Complementary tests, including chest and right pelvic limb radiography and tomography, were performed (Figure 1).

The radiograph of the right pelvic limb revealed an aggressive bone response, characterized by a geographic area mainly composed of osteolysis with poorly defined margins, cortical thinning and destruction, and amorphous, spiculated periosteal proliferation, extending from the distal metaphyseal region to the proximal diaphyseal region of the femur. This lesion exhibited a mixed pattern of lytic and proliferative characteristics, resulting in significant loss of cortical definition, which is indicative of bone matrix lysis and invasion into the spongy bone and endosteum. The lesion was located in the distal diaphysis of the right femur and extended to its distal metaphysis in the medial cranial aspect. Additionally, apparent atrophy present in the biceps femoris, semimembranosus and gracilis muscles suggest limb disuse or lameness. No lesions were observed in the femoral condyles.

Thoracic radiographs showed no evidence of metastasis. Samples from the inguinal lymph node and the mass in the distal region of the femur predominantly consisted of the same cell groups as those found in the mammary mass. The diagnosis was confirmed as malignant mammary neoplasm with metastasis to the inguinal lymph node and to the distal region of the femur. A unilateral partial mastectomy (M3 to M5) was performed on the left side, along with a high amputation of the right pelvic limb and lymphadenectomy of the corresponding regional lymph nodes (right and left inguinal). The excised tissue was submitted for histopathological examination.

The second dog (case 02) was a 9-year-old, non-spayed mixed-breed female with a history of exogenous progesterone use. The owner reported the appearance of nodules three months prior, which had progressively grown and ulcerated. During the clinical examination, the dog was prostrated. A chest radiograph revealed a lesion suggestive of pulmonary metastasis. Due to the poor prognosis and the animal's clinical condition, euthanasia was performed in October 2018.

During necropsy, external examination revealed a friable mass measuring 8.0 x 7.0 cm in the right mammary chain, located cranially to the caudal thoracic mammary gland (M2). When incised, the mass exhibited liquefied consistency and released abundant amount of red fluid with a putrid odor. A plaque-like mass measuring 10.0 x 6.5 cm was observed in the cranial (M3) and caudal (M4) abdominal mammary glands. Upon sectioning, extensive cystic areas with whitish, pasty content were noted. The inguinal mammary gland (M5) was extensively ulcerated, with the ulcer measuring approximately 15.0 x 11.0 cm.

Internal examination revealed multifocal yellowish nodules ranging from 0.1 to 1.0 cm on the liver, multifocal whitish nodules of similar size on the kidneys, and an orange nodule measuring 0.8 cm on the adrenal cortex, with a white nodule measuring 1.0 cm in the medullary region. The heart exhibited multifocal whitish areas of 0.1 cm in the endocardium; whitish multifocal to coalescent nodules with average size of 0.3 cm were found in the lungs, primarily in the caudal lobes. The medial iliac lymph node was markedly enlarged and firm, with a whitish, homogeneous parenchyma upon sectioning. The right axillary lymph node measured 6.0 x 4.0 cm, was firm in consistency, and showed complete loss of corticomedullary distinction on sectioning. No alterations were observed in the other organs during macroscopic examination.

**Figure 2:**
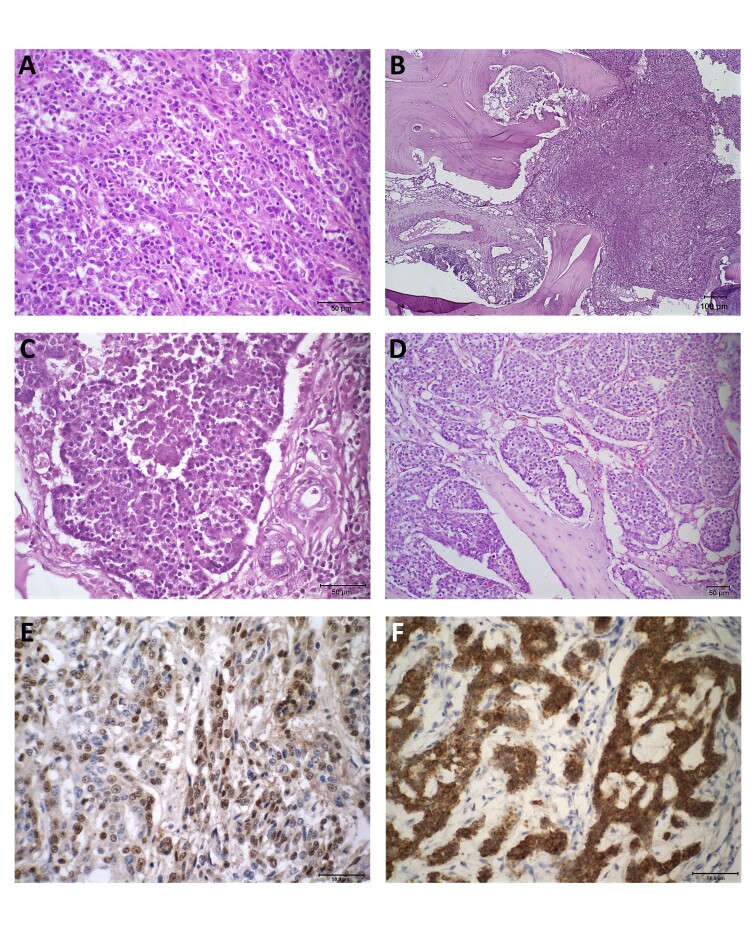
Microscopy. Bitch. Mammary gland. Hematoxylin and eosin (HE) staining reveals epithelial and myoepithelial cells organized into solid nests (A) HE shows epithelial and myoepithelial cells arranged in solid nests in the femur of case 1 (B). HE shows proliferation of a population of neoplastic epithelial cells, forming a sieve-like arrangement in case 2 (C). HE shows proliferation of a population of neoplastic epithelial cells in the femur (D). Immunohistochemical staining showing positivity for pan-cytokeratin (E) and p63 (F) in the primary tumor of case 1, confirming the diagnosis of malignant adenomyoepithelioma.

Post-mortem radiographs of the thoracic and pelvic limbs were evaluated, revealing a moderately aggressive bone response exclusively in the proximal metaphyseal region of the right humerus, extending to its distal diaphyseal region. These changes were characterized by poorly defined medullary bone sclerosis, interrupted fine brush-like periosteal proliferation, and cortical thinning and destruction. A longitudinal section of the humerus revealed a focally extensive whitish-brown area involving the periosteum and the spongy portion of the bone. The primary necropsy findings are illustrated in Figure 2. The tissue samples were processed with routine techniques, including paraffin embedding, hematoxylin and eosin staining, and analysis under an optical microscope.

### Histopathological evaluation

The histopathological evaluation of the primary tumor of the first animal (case 01) showed dense neoplastic cellular proliferation, composed of myoepithelial and epithelial cells, formed by varied arrangements, and presenting papillary, tubular, and solid nest proliferations. Myoepithelial cells exhibited slightly fusiform and vacuolized cytoplasm, with high nucleus/cytoplasm ratio, ovoid nuclei, finely granular chromatin, and prominent nucleoli. Carcinomatosis cells were polyhedral, with vacuolized cytoplasm, high nucleus/cytoplasm ratio, ovoid nuclei, and prominent nucleoli (Figures 2A and 2B). Also, moderate anisokaryosis and endocytosis were observed. In general, the neoplasms presented high mitotic count, with an average of 21 mitosis figures in 10 high power fields (40x, 2,37mm^2^). Such morphological characteristics are consistent with the diagnosis of adenomyopethelioma (Cassali et al., 2020; Meuten et al., 2017; Sorenmo et al., 2013), with metastases in the analyzed lymph nodes and bones. The mass in the distal region of the femur was characterized by multilobular neoplastic cell proliferation, which infiltrated the musculature and adjacent bone tissue with characteristics similar to those observed in the mammary gland’s mass.

The histopathological evaluation of the primary tumor in M3, M4 and M5 of the second animal (case 02) showed dense neoplastic cellular proliferation composed of epithelial cells in a cribriform arrangement characterized by the formation of nodules with pseudo-lumens in the periphery and necrotic center. Epithelial cells had reduced eosinophilic cytoplasm with indistinct borders, oval nuclei, sparse chromatin and evident, sometimes multiple nucleoli. Moderate anisocytosis and anisokaryosis and three mitotic figures were observed in 10 high-power fields (400x, 2.37 mm^2^). Therefore, the histopathological diagnosis was grade II cribriform carcinoma (Cassali et al., 2020; Meuten et al., 2017; Sorenmo et al., 2013). The histopathological evaluation of the bone metastasis of the second animal (case 02) showed that the histological section of the humerus presented solid nests of epithelial cells with marked atypia and high mitotic activity with bizarre mitoses, interspersing bone trabeculae, characterizing bone metastasis. Atypical epithelial cells were also found in a lung, liver, kidney, adrenal and axillary and medial iliac lymph node’s parenchyma. Neoplastic emboli were also noted in the intestines and uterus.

Histological sections of the primary tumors and bone metastasis of 3 μm of thickness were prepared and mounted on common slides for immunohistochemistry analysis. The antigen was immunodetected using the detection system anti-mouse/anti-rabbit (Novolink Polymer Detection System, Leica Biosystems, Newcastle Upon Tyne, Reino Unido) according to the manufacturer’s instructions. The endogenous peroxidase activity was blocked with a 10% hydrogen peroxide (H_2_O_2_) solution in methyl alcohol. Reagents were manually applied and immunoreactivity was visualized by incubation of the slides with chromogen diaminobenzidine (DAB Substrate System, Dako, Carpinteria, CA, USA) for 3 minutes. Details of the antibodies, dilutions, antigen retrieval procedures and incubation times used in the immunostaining process are shown in Table 1. Details regarding this analysis (Table 1).

**Table 1:** Target antigens and clones, dilutions, antigen retrieval methods, and incubation times and temperatures for immunohistochemical staining for Ki-67, hormone receptors (estrogen receptor, ER, progesterone receptor, PR), human epidermal growth receptor 2 (HER-2), transformation-related protein 63 (p63), pancytokeratin (pan-CK) and Cox-2.

**Target Antigen (Clone)**	**Dilution**	**Antigen Retrieval Method**	**Incubation Time (h)/Temp**
**Ki-67**	1:50	Pressurised Heat (125 °C/2min) with citrate buffer pH 6.0	14-16h/4 °C
**(MIB-1)**			
**ER**	1:50	Pressurised Heat (125 °C/2min) with citrate buffer pH 6.0	14-16h/4 °C
**(1D5)**			
**PR**	1:50	Pressurised Heat (125 °C/2min) with citrate buffer pH 6.0	14-16h/4 °C
**(hPRA2)**			
**HER-2 (Polyclonal)**	1:200	Double boiler (95 °C/20min)with citrate buffer pH 6.0	14-16h/4 °C
**P63**	1:100	Pressurised Heat (125 °C/2min) with citrate buffer pH 6.0	14-16h/4 °C
**(DAK-p63)**			
**Pan-CK**	1: 500	Water bath (98 °C/20min) with citrate buffer pH 6.0	14-16h / 4 °C
**(AE1/AE3)**			
**Cox-2**	1:50	Pressurised Heat (125 °C/2min) with citrate buffer pH 6.0	14-16h / 4 °C
**(SP21)**			

For Ki-67 evaluation, at least 1,000 neoplastic cells in high-power (400X) fields were analyzed, whereas nuclear labeling </= 20% was considered a low proliferation rate and >20% was considered a high proliferation rate. For ER and PR evaluation, >10% nuclear labeling was considered positive. Cox-2 was evaluated based on staining intensity (graded as weak [1], moderate [2], or strong [3]) and the percentage of labeled cells (<10% [1], 10-30% [2], 31-60% [3], >60% [4]). The staining score is given from the multiplication between the staining intensity and the percentage of labeled cells, ranging from 0, negative, to 12, strongly positive. A scoring system established by Nunes et al, 2022 was used to determine HER2 expression: 0 = no membrane staining or incomplete and faint/barely perceptible membrane staining in ≤10% of tumor cells; 1+ = incomplete and faint/barely perceptible membrane staining in ≥10% of tumor cells; 2+ = incomplete and/or weak/moderate membrane staining in >10% of tumor cells or complete and intense membrane staining in ≤10% of tumor cells; and 3+ = complete and intense membrane staining in >10% of tumor cells). Specimens with scores of 0, 1+ and 2+ were regarded as negative, whereas a score of 3+ was defined as positive. Pancytokeratin was positive for any percentage of membrane staining, while p63 was positive for any percentage of nuclear staining. The immunophenotype of the primary tumors and bone metastases was classified as luminal B, following the criteria established by Nunes et al. (2022). The results are detailed in Table 2.

**Table 2:** Immunohistochemical results for Ki-67, hormone receptors, human epidermal growth receptor 2 (HER-2), transformation-related protein 63 (p63), pancytokeratin (pan-CK) and Cox-2 in primary tumor and bone metastasis.

**Target Antigen**	**Primary tumor**	**Bone metastasis**	**Primary tumor**	**Bone metastasis**
	(case 1)	(case 1)	(case 2)	(case 2)
**Ki-67**	73,8%	92,6%	87,3%	94,7%
**ER**	26-50% (++)	26-50% (++)	26-50% (++)	26-50% (++)
**PR**	>75% (++++)	>75% (++++)	>75% (++++)	>75% (++++)
**HER-2**	+	+	++	++
**P63**	Positive	Positive	Positive	Positive
**Pan-CK**	Positive	Positive	Positive	Positive
**Cox-2 (SP21)**	Negative	Negative	Negative	6

Meaning of (+) and (++) for HER-2: ssee description of materials and methods.

## Discussion

Bone metastases are uncommon in dogs with tumors, but this incidence may be underestimated (Cooley & Waters, 1998), as imaging exams are not routinely performed to detect distant metastases. Additionally, after death, many owners do not opt for necropsies due to the costs involved, and even when necropsies are conducted, the skeletal system is often not thoroughly examined for bone metastases. These factors contribute to the underreporting and difficulty in determining the true frequency of bone metastases from mammary carcinomas in bitches.

Typically, the lungs are the most common site of distant metastasis. Other sites of metastasis include sternal, sublumbar, and prescapular lymph nodes, liver, brain, and bones (Cassali et al., 2020). While bone metastases have been reported to be preceded by metastases in other organs (Cooley & Waters, 1998), they can also occur without prior or concomitant lung metastases (Trost et al., 2014). Bone metastasis can manifest as osteolytic (bone resorption) or osteoblastic (bone formation) lesions, leading to pain, fractures, and increased mortality. The general pathogenesis of bone metastasis involves the proliferation of the primary tumor, local tissue invasion, intravasation into blood vessels, extravasation into the bone marrow, a variable period of tumor cell dormancy, proliferation within the bone, and modification of the bone microenvironment (Fidler, 2003).

Radiographically, the cases presented in the present study demonstrated classic features of bone metastasis. The radiographs revealed aggressive bone responses, including areas of osteolysis with poorly defined margins, significant cortical thinning, and destruction. The presence of amorphous, spiculated periosteal proliferation was particularly noted, extending from the metaphyseal to the diaphyseal regions of the affected bones. These radiographic findings are consistent with those typically observed in bone metastases, where the aggressive nature of the lesions indicates a highly destructive process, often leading to substantial bone weakening and potential for pathological fractures. The mixed lytic and proliferative patterns seen in these radiographs further highlight the complex interaction between tumor cells and the bone microenvironment, with bone resorption and abnormal new bone formation occurring simultaneously (Fornetti et al., 2018; Hiraga, 2019; Kakhki et al., 2013; Weilbaecher et al., 2011).

The skeleton, composed of dynamic tissue, plays a critical role in support and movement, besides serving as a reservoir for minerals and energy. Bones also house the bone marrow, the primary site of hematopoiesis (Fornetti et al., 2018). Bone tissue is composed of various resident cells, most notably osteoblasts and osteocytes, which maintain structural integrity and bone homeostasis, and osteoclasts, which regulate the bone remodeling process in response to mechanical stimuli and systemic hormones (Fornetti et al., 2018; Sims & Martin, 2014). The anatomical structure of bones, which includes osteoblasts, osteoclasts, bone lining cells, and osteocytes, contributes to bone homeostasis (Bonewald, 2011; Fornetti et al., 2018; Sims & Martin, 2014).

The current understanding of the preferential localization of cancer cells in bone is based on Paget's “seed and soil” hypothesis, which suggests neoplastic cells (seeds) proliferate only in a suitable environment, such as bone marrow (Paget, 1989). However, recent studies have shown that the bone marrow endothelium, adipocytes, and the immune environment also play a role in bone tissue homeostasis (Fornetti et al., 2018; Hiraga, 2019; Yip et al., 2021). Although bone metastasis is common in many solid tumors, not all bones harbor metastases, suggesting that bone colonization by tumor cells is not random and/or that not all bones provide a conducive environment for metastasis establishment and growth. For example, metastases are often found in bones rich in red marrow and trabecular bone, such as the vertebrae, ribs, pelvis, and ends of long bones, but are rarely seen in bones of the hands or feet (Fornetti et al., 2018; Krishnamurthy et al., 1977). This pattern was observed in the two cases presented in this report. The selectivity is not well understood, but the presence of trabecular bone, higher rates of bone turnover, and increased vascularity at frequent sites of bone metastasis could contribute to tumor cell growth (Fornetti et al., 2018). The bone matrix also plays a crucial role in the maintenance of bone tissue and the metastasis of mammary cancer cells to the bone. In addition to providing structural support for cells residing in bone, the bone matrix contains a multitude of growth factors that are released during normal bone turnover (Fornetti et al., 2018; Lind, 1998). These same factors can also fuel the growth of metastatic tumor cells in the skeletal system (Fornetti et al., 2018).

Immunohistochemical evaluation of the two cases revealed a high proliferative rate, as indicated by Ki67 immunostaining in more than 20% of neoplastic cells. This feature is associated with an unfavorable prognosis and an increased likelihood of distant metastases (Cassali et al., 2020; Nunes et al., 2022). In both cases, HER-2 was negative, and hormone receptors were positive in both the primary tumors and bone metastases. This classifies the molecular subtype of the primary tumors and bone metastases as luminal B, consistent with findings from a study by Gerratana et al. (2015), which evaluated 544 patients with metastatic breast cancer. The study found that bone was the most frequent site of metastasis for mammary gland carcinomas positive for hormone receptors (luminal subtypes), while triple-negative molecular subtypes were the least frequent. Among the luminal subtypes, luminal B was the most common molecular subtype (Gerratana et al., 2015).

In addition to the histopathological characteristics, the diagnosis was confirmed by positive immunostaining for Pan-CK, an immunomarker for epithelial cells, and p63, an immunomarker for myoepithelial cells, in the canine mammary gland (Cassali et al., 2020). To confirm that the cells in the bone tissue sample originated from the mammary gland and to verify bone metastasis, positive immunostaining for hormone receptors, Pan-CK, and p63 was essential. Finally, COX-2 was negative in the primary tumors of both cases, negative in the metastasis of case 01, and positive in the metastasis of case 02.

## Conclusion

Mammary tumors are the most common neoplasms in bitches and often exhibit aggressive behavior and potential for metastasis. Bone metastasis, though rare, may be underdiagnosed due to limited imaging and post-mortem examinations. The present study highlights the importance of comprehensive diagnostics, including imaging and histopathological analysis, to accurately identify metastatic sites. Immunohistochemical profiling proves essential for prognosis and therapeutic guidance, aiding in the identification of tumor subtypes and metastatic potential. These findings contribute to a better understanding of canine mammary carcinomas, supporting advancements in
